# Etymologia: *Capnocytophaga canimorsus*

**DOI:** 10.3201/eid2412.ET2412

**Published:** 2018-12

**Authors:** Ronnie Henry

**Keywords:** Capnocytophaga canimorsus, bacteria, gliding bacteria, dogs, cats, carbon dioxide, oral microbiota, septicemia, meningitis

## *Capnocytophaga canimorsus* [kapʺno-si-tofʹǝ-gǝ kanʺǝ-morʹsǝs]

From the Greek *kapnos* (“smoke”) for its dependence on carbon dioxide, which is a large component of smoke, *Capnocytophaga canimorsus* (Latin *canis*, “dog,” and *morsus*, “bite”) are gram-negative, facultatively anaerobic, rod-shaped bacteria that are part of the normal oral microbiota of dogs and cats (Figure). The genus was proposed to distinguish these bacteria from *Cytophaga* spp. (Greek *kytos*, “cell,” and *phagein*, “eat”), which also exhibit gliding motility. *C. canimorsus* was previously known as CDC group DF-2 (dysgonic fermenter type 2) and was first isolated from a man who had experienced multiple dog bites and developed septicemia and meningitis. *C. canimorsus* remains a major cause of septicemia in persons, particularly those who are asplenic or immunocompromised, who are bitten by dogs or cats.

**Figure:**
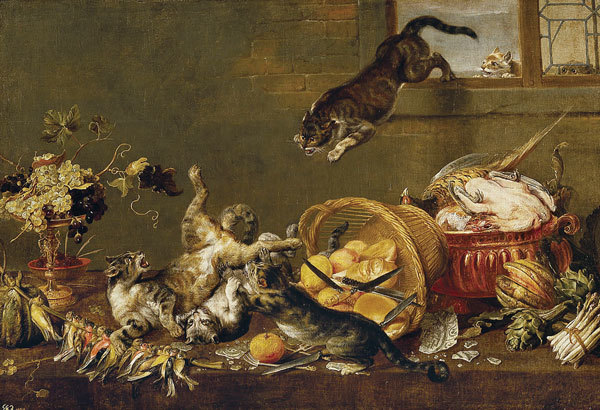
**Paul de Vos, Cats Fighting in a Larder 1630–1640.** Oil on canvas. Museo Nacional del Prado. https://www.museodelprado.es/coleccion/galeria-on-line/galeria-on-line/obra/pelea-de-gatos-en-una-despensa/, Public Domain, https://commons.wikimedia.org/w/index.php?curid=39117357
